# Short-Term Morpho-Functional Changes before and after Strabismus Surgery in Children Using Structural Optical Coherence Tomography: A Pilot Study

**DOI:** 10.3390/vision8020021

**Published:** 2024-04-16

**Authors:** Pasquale Viggiano, Marida Gaudiomonte, Ugo Procoli, Luisa Micelli Ferrari, Enrico Borrelli, Giacomo Boscia, Andrea Ferrara, Fabio De Vitis, Gemma Scalise, Valeria Albano, Giovanni Alessio, Francesco Boscia

**Affiliations:** 1 Department of Translational Biomedicine Neuroscience, University of Bari “Aldo Moro”, 70125 Bari, Italy; maridagaudiomonte@gmail.com (M.G.); luisa.micelliferrari@gmail.com (L.M.F.); bosciagiacomo@gmail.com (G.B.); ferrarandrea@hotmail.com (A.F.); devitisfabio@gmail.com (F.D.V.); gemma.scalise@libero.it (G.S.); valeria.albano12@gmail.com (V.A.); giovanni.alessio@uniba.it (G.A.); francescoboscia@hotmail.com (F.B.); 2 Department of Surgical Sciences, University of Turin, 10124 Turin, Italy; borrelli.enrico@yahoo.com; 3 Department of Ophthalmology, “City of Health and Science” Hospital, 10124 Turin, Italy

**Keywords:** strabismus, OCT, macular ganglion cell–inner plexiform layer

## Abstract

Purpose: To evaluate the immediate alterations in the thickness of the macular ganglion cell–inner plexiform layer (mGCIPL), peripapillary retinal nerve fiber layer (RNFL), inner retinal layer (IRL), and outer retinal layer (ORL) using spectral domain optical coherence tomography (SD-OCT) subsequent to strabismus surgery in pediatric patients diagnosed with horizontal esotropia. Methods: Twenty-eight eyes from twenty-one child patients who had undergone uncomplicated horizontal rectus muscle surgery due to strabismus were included. Measurements of RNFL, mGCL-IPL, IRL, and ORL using structural OCT were conducted both before the surgery and one month after the surgical procedure. Importantly, a control group comprising 14 healthy eyes, matched for age and significant refractive error (<3.00 diopters), was included in the current analysis. Results: Our analysis indicated no significant disparity before and after surgery in terms of best-corrected visual acuity (BCVA), RNFL, IRL, and ORL. Conversely, concerning the macular ganglion cell layer–inner plexiform layer analysis, a substantial increase in mGCL-IPL was observed following the surgical intervention. The mean mGCL-IPL measured 60.8 ± 9.2 μm at baseline and 66.1 ± 13.2 μm one month after the surgery (*p* = 0.026). Notably, comparison between the strabismus group at baseline and the healthy group revealed a significant reduction in mGCL-IPL in the strabismus group (60.8 ± 9.2) compared to the healthy control group (68.3 ± 7.2; *p* = 0.014). Conclusions: Following strabismus surgery, our observations pointed towards a thickening of the mGCL-IPL layer, which is likely attributable to transient local inflammation. Additionally, we identified a significant differentiation in the mGCL-IPL complex between the pediatric patient group with strabismus and the control group.

## 1. Introduction

Strabismus is an ocular condition characterized by the failure to maintain parallelism in the visual axes of both eyes while focusing on an object. This condition typically begins in childhood and can result in double vision (diplopia), reduced vision in one eye (amblyopia), and a loss of the ability to perceive depth and dimension (binocularity) [1]. Strabismus is a prevalent eye condition that can have psychological implications for both children and their parents. Studies indicate that the occurrence of strabismus in children fluctuates globally between 2% and 6% among diverse ethnic groups [2,3,4,5].

Treatment options for strabismus in patients vary depending on the underlying cause and may involve observation with regular follow-up, as well as medical and surgical interventions. Surgical treatment for strabismus includes various procedures that aim to weaken the muscle, such as marginal myotomy, disinsertion, myectomy, graded recession, anterior transposition, and nasal myectomy [6,7]. Importantly, studies have demonstrated that performing strabismus surgery before visual maturity, typically around 9 years of age, can yield potential functional advantages [7,8].

The recent focus has been on whether extraocular muscle surgery impacts the macular region [9,10]. Research has observed alterations in the macula and choroid during the initial phase, although the findings are inconsistent. Some studies have documented an elevation in macular thickness, while others have reported no discernible changes [11,12]. Improved imaging techniques, notably optical coherence tomography (OCT), have revealed associations between vision impairment and the peripapillary retinal nerve fiber layer (RNFL), macular thickness, as well as alterations in retinal layering and the retinal vascular structure [13,14,15]. Optical coherence tomography is an interferometric technique that generates a cross-sectional view of the retina based on the reflectivity of different layers within the retina. It is widely utilized for macular examination, being the sole imaging modality that permits direct visualization of the layered retinal structures [16]. The advancements in OCT technology have introduced an objective and quantitative approach for assessing macular thickness and volume.

However, there is a dearth of research examining changes in the neuroretinal layers, including the RNFL and the macular ganglion cell–inner plexiform layer (mGCIPL), following strabismus surgery. Thus, the aim of this current study is to evaluate the short-term changes in the thickness of the mGCIPL, RNFL, inner retinal layer (IRL), and outer retinal layer (ORL) using spectral domain OCT (SD-OCT) following strabismus surgery for pediatric patients with horizontal esotropia.

## 2. Materials and Methods

This retrospective cohort study received approval by our local ethics committee. The study adhered to the principles outlined in the 1964 Helsinki Declaration, as well as its subsequent amendments. We reached out to all enrolled individuals and their parents to secure their written informed consent for the retrospective utilization of their clinical information.

This study involved the imaging of 28 eyes from 21 patients, ranging in age from 5 to 10 years, who were affected by esotropia deviation, utilizing SD-OCT (Heidelberg Engineering, Heidelberg, Germany). Preoperative and postoperative (1 month after the surgical procedure) measurements of refractive error, fundus examination, intraocular pressure measurements, and SD-OCT were conducted. Best-corrected visual acuity (BCVA) was determined using Snellen charts and subsequently converted into logarithm of the minimal angle of resolution (LogMAR) equivalents. The assessment of ocular deviations was conducted using the prism and cover test during the examination preceding the strabismus surgery. 

Furthermore, a control group comprising 14 healthy eyes, matched for age, significant refractive error (<3.00 diopters), and ethnicity, was included in the current analysis. The mean spherical equivalent (SE) value was −1.21 ± 1.92 diopters (D), with a range of −2.25 to 1.75 D in the control group.

The study excluded patients with systemic diseases such as diabetes mellitus and neurological conditions, those with pre-existing ocular issues including macular pathology and corneal disorders, individuals with a history of prior ocular surgery, as well as those with a refractive error exceeding 3.00 diopters. Additionally, participants unable to undergo SD-OCT and those who did not attend regular follow-up appointments after the surgical procedure were also excluded.

### 2.1. Surgical Procedure

All surgical procedures were conducted by the same surgeon (UP) under general anesthesia. The medium rectus muscle surgeries were performed via limbal incision, and the recession was performed with the hang-back technique. The recessed muscle was then sutured to the sclera using 6–0 Vicryl sutures. Subsequently, the conjunctiva was sutured with 8–0 Vicryl sutures, and the surgery was concluded. Postoperatively, patients received a regimen of topical antibiotic, steroid, and artificial eye drops for a duration of 2 weeks.

### 2.2. Imaging Protocol

A retinal assessment was performed utilizing SD-OCT imaging with the Spectralis OCT from Heidelberg Engineering, Inc, Heidelberg, Germany. During each examination, volumetric scans were focused on the fovea, consisting of 97 horizontal B-scans, each of which was composed of 18 averaged scans, covering a 5.8 mm × 5.8 mm region centered on the fovea [17]. The final visit was conducted in accordance with a tracking progression. B-scans were separated by intervals of 60 microns. Any images of inadequate quality, characterized by a signal strength below 25, were excluded and substituted with fresh scans as required.

### 2.3. Outcomes Measured

**Figure 1:**
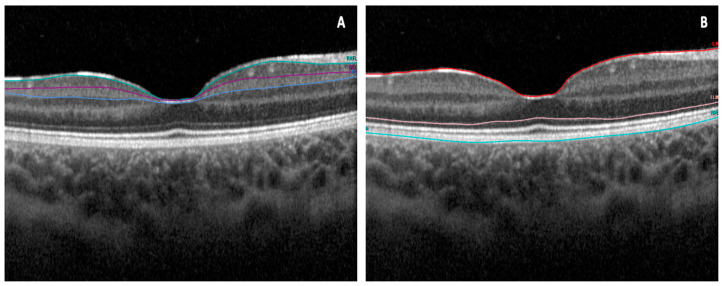
Illustrates (**A**) an instance of automated segmentation of the retinal nerve fiber layer, ganglion cell layer, and inner plexiform layer using Heidelberg Spectralis. (**B**) Additionally, it shows an example of automated segmentation of the inner retinal and outer retinal layers.

This study evaluated several tomographic parameters, such as the macular ganglion cell layer (mGCL), macular inner plexiform layer (mIPL), and macular RNFL thickness (mRNFL) (Figure 1A). Additionally, it analyzed the IRL, extending from the inner limiting membrane to the external limiting membrane, and the ORL, spanning from the external limiting membrane to Bruch’s membrane (Figure 1B), utilizing the integrated automated software Spectralis (Heyex 2). 

**Figure 2:**
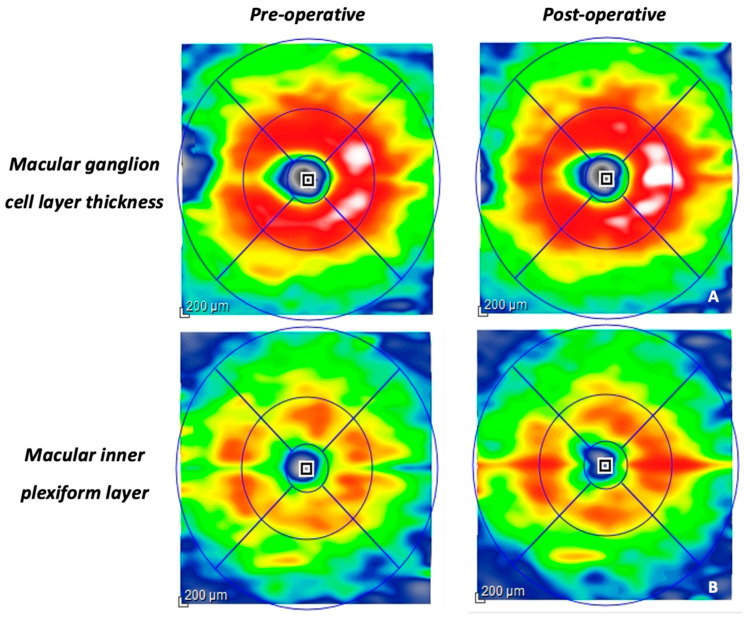
Illustrates (**A**) a colorimetric map of the ganglion cell layer with ETDRS circle diameters (1, 3, 6 mm) before and after the strabismus surgery. (**B**) Additionally, it showcases the changes in the inner plexiform layer before and after the surgical procedure.

The combined measurement of the mGCL-IPL was computed by incorporating the mGCL and mIPL parameters [18]. Prior to calculating the thickness values, the graders meticulously examined all B-scans and manually rectified any segmentation or decentration errors. Measurements of mRNFL, mGCL-IPL, IRL, and ORL using structural OCT were conducted both before the surgery and one month after the surgical procedure (Figure 2A,B). 

### 2.4. Statistical Analysis 

To assess deviations from a normal distribution, a Shapiro–Wilk’s test was conducted for all variables, and the results confirmed that the data met the assumptions of normality. For the comparison of best-corrected visual acuity and OCT parameters between baseline and 1 month after the surgical procedure, a paired *t*-test was employed. A Student’s *t*-test was used to compare BCVA and OCT parameters between the strabismus group and the healthy control group. A *p*-value of less than 0.05 was considered statistically significant. Statistical analysis was performed using the Statistical Package for Social Sciences (version 23.0, SPSS Inc., Chicago, IL, USA), with the predetermined level of statistical significance set at *p* < 0.05.

## 3. Results

### 3.1. Characteristics of Patients Included in the Analysis

This study involved a total of 28 eyes belonging to 21 patients diagnosed with strabismus. Among the patients, 18 were male, and 3 were female. Additionally, a control group comprising 14 eyes was also included in the study. The mean age of the patients in the strabismus group was 7.23 ± 1.3 years (range: 5–10 years), while the mean age of the control group was 7.14 ± 1.2 years (range: 5–10 years). The comparison between the two groups did not reveal any significant differences (*p* ≥ 0.05). Details of the subjects who were included in the analysis are provided in Table 1. Subjects with strabismus were assessed both before the surgery (the day before the surgical procedure) and one month after the surgical procedure. The outcome measures included the BCVA, as well as measurements of the mRNFL, mGCL-IPL, IRL, and ORL.

### 3.2. Outcome Analysis in Strabismus Group 

BCVA (logMAR) analysis revealed a mean ± SD BCVA value of 0.1 ± 0.3 logMAR at baseline and 0.0 ± 0.2 logMAR one month after the surgical procedure (*p* = 0.089).

In the analysis of the macular retinal nerve fiber layer, the mean global mRNFL thickness before the surgical procedure was 15.2 ± 1.8 μm, which increased to 16.1 ± 2.8 μm one month after the surgery. However, this difference was not found to be statistically significant (*p* = 0.121).

For the macular ganglion cell layer–inner plexiform layer analysis, the mGCL-IPL was observed to have significantly increased after the surgical procedure. The mean mGCL-IPL was 60.8 ± 9.2 μm at baseline and 66.1 ± 13.2 μm one month after the surgery (*p* = 0.026). Regarding the inner retinal layer thickness analysis, the mean IRL thickness before the surgical procedure was 215.1 ± 18.9 μm, which slightly increased to 219.2 ± 25.3 μm after the surgery. However, this difference was not statistically significant (*p* = 0.221).

In the outer retinal layer thickness analysis, the mean ORL thickness before the surgical procedure was 84.3 ± 3.6 μm, which increased to 84.9 ± 2.7 μm after the surgery. Similar to the previous analyses, this difference was not statistically significant (*p* = 0.669) (Table 2). 

### 3.3. Comparison between Groups 

Significantly, our comparison between the strabismus group at baseline and the healthy group revealed that the mGCL-IPL was notably reduced in the strabismus group (60.8 ± 9.2) in comparison to the control healthy group (68.3 ± 7.2; *p* = 0.014). However, regarding the BCVA, mRNFL, IRL, and ORL, we did not identify any significant changes (*p* ≥ 0.05) (refer to Table 3). Furthermore, the comparison between the post-surgery strabismus group and the healthy group did not reveal significant differences in terms of the BCVA, mGCL-IPL, mRNFL, IRL, and ORL (Table 4).

## 4. Discussion

OCT is a noninvasive method used for high-resolution cross-sectional tomographic imaging of the retina and optic nerve head [13]. It is the most frequently utilized imaging technique for conducting macular examinations. Notably, OCT can identify retinal thickening even when no abnormality is observed during a slit lamp examination. With the progression of OCT technology, it has become possible to acquire quantitative neuroretinal parameters, including mRNFL and a quantification of ganglion cells [18]. 

Research on the impact of strabismus surgery on the macula remains limited and has produced varied results. With the intention of contributing to the existing literature, the present study aimed to assess the morphological alterations in the mRNFL, mGCL-IPL, IRL, and ORL thicknesses after rectus muscle surgery. Notably, we observed a notable increase in the mGCL-IPL following strabismus surgery in pediatric patients. It is important to highlight that our analysis indicated a significant reduction in the mGCL-IPL in the strabismus group before the surgery when compared to the healthy control eyes. 

Research conducted on animals has demonstrated that visual deprivation can lead to structural changes in the retina, such as the degeneration of retinal ganglion cells (RGCs) [19,20], a decrease in the nucleolar volume and cytoplasmic cross-sectional area of RGCs [21,22], an increase in the number of amacrine synapses in the IPL, a reduction in the number of bipolar synapses in the IPL [23], and a reduction in the density of Müller fibers [24].

Indeed, the mRNFL, mGCL, and mIPL encompass the axons, cell bodies, and dendrites of the retinal ganglion cells. One potential explanation of our findings is that the strabismus group exhibits a decreased cellular and dendritic density in the mGCL and mIPL compared to the healthy group [25]. Some studies suggest that reduced metabolism due to a decrease in visual stimuli reaching the impaired eye might contribute to these differences [25]. Moreover, the retinal synaptic pathway and retinogeniculate synapses undergo postnatal remodeling, influenced by visual stimulation [26]. Further, recent evidence from histological and in vivo OCT studies indicates that foveal development extends beyond early childhood (until 5 years old) and persists into the teenage years, suggesting that prolonged sensory deprivation could significantly impact retinal structural changes [27]. The age range of our study cohort comprised pediatric patients aged between 5 and 10 years old, which may further emphasize the initial distinctions between the two groups. Additional potential factors contributing to the divergent outcomes could include the challenge in controlling the severity of the visual impairment or the duration of the visual deprivation.

In a study conducted by Park et al. [28], wherein the thickness of each retinal layer was measured, significant thinning of the mGCIPL was observed in eyes with various types of visual impairment, including cases of strabismus, aniso-astigmatism, and unilateral ptosis. This finding is consistent with the results of Xia et al. [29], who conducted an analysis of anisometropic amblyopia and identified a significant reduction in the thickness of two out of the three GCL quadrants and three out of the four IPL quadrants in the peripheral macular area compared to the control group.

However, our post-surgical findings indicate a thickening of the mGCIPL. This observation might be attributed to the edema resulting from the inflammation of the cell bodies and dendrites of the nerve layers as a response to the surgical procedures performed. Indeed, it has been proposed that surgical trauma can result in the release of prostaglandins in the aqueous humor, leading to the impairment of the blood–aqueous barrier. This disruption may facilitate the accumulation of various inflammatory mediators, such as endotoxins and immune complexes, in the aqueous humor [30].

Mintz et al. [9] and Guler Alis et al. [31] reported an increase in the central macular thickness in patients who underwent horizontal rectus muscle surgery. This finding partially aligns with the outcomes of our study. Nevertheless, we conducted a subanalysis of the neuroretinal layers to gain a deeper understanding of which specific retinal layers were primarily affected by post-surgical alterations. We hypothesize that the observed thickening of the mGCL-IPL could potentially be attributed to a transient local inflammation following the traumatic intervention, even though the retina itself was not directly impacted. Studies with a larger patient cohort and longer follow-up durations will be necessary to determine whether this mGCL-IPL thickening is temporary or indicative of a more permanent change.

Lastly, we did not identify any notable changes in terms of the BCVA, mRNFL, IRL, and ORL’s thickness before and after the surgery.

Our study is not without limitations. The primary limitation of our study is the small sample size, which may limit the generalizability of the results. Moreover, the retrospective nature of the study and the lack of assessment of biometric parameters are the most significant constraints. Another important limitation of our study was the analysis of both eyes in some enrolled patients. Furthermore, the surgical procedure involved the recession of only one rectus muscle per eye, which may have significantly influenced our results. Additionally, to conduct a proper statistical analysis, a comparison with the post-surgery control group data of the group affected by strabismus should have been performed.

In conclusion, our study revealed a notable distinction in the mGCL-IPL complex between the group of pediatric patients with strabismus and the control group. Strabismic children exhibited a decreased thickness of the mGCL-IPL complex. Moreover, post strabismus surgery, our findings indicated a thickening of the mGCL-IPL layer, likely as a result of transient local inflammation. We believe that more precise information can be obtained through studies conducted on more homogeneous groups with a larger number of patients and longer follow-up durations.

## Figures and Tables

**Table 1:** Demographic and clinical description of child patients with strabismus and children from control group.

	Strabismus Group	Control Group	*p* Value
Number of patients, *n*	21	14	0.217
Number of eyes, *n*	28	14	0.175
Age (*years*)	7.23 ± 1.3	7.14 ± 1.2	0.989
Gender (*M⁄F*)	18/3	9/5	0.345
Initial BCVA (*logMAR*)	0.1 ± 0.3	0.0 ± 0.0	0.679

Quantitative values are expressed in mean ± SD (standard deviation). *BCVA*—best-corrected visual acuity; *logMAR*—logarithm of the minimum angle of resolution.

**Table 2:** Morpho-functional results in strabismus group. Data and comparisons.

	Before Surgical Procedure	1 Month after Surgical Procedure	*p* Value
BCVA (logMAR)	0.1 ± 0.3	0.0 ± 0.2	0.089
RNFL (μm)	15.2 ± 1.8	16.1 ± 2.8	0.121
mGCL-IPL (μm)	60.8 ± 9.2	66.1 ± 13.2	0.026
IRL (μm)	215.1 ± 18.9	219.2 ± 25.3	0.221
ORL (μm)	84.3 ± 3.6	84.9 ± 2.7	0.669

Quantitative values are expressed in mean ± SD (standard deviation). BCVA—best-corrected visual acuity; RNFL—retinal nerve fiber layer; mGCL-IPL—macular ganglion cell layer–inner plexiform layer; IRL—inner retinal layer; ORL—outer retinal layer. *Paired test was performed to obtain p-values.*

**Table 3:** Morpho-functional comparison between strabismus group and control group.

	Strabismus Group at Baseline	Control Group	*p* Value
BCVA (logMAR)	0.1 ± 0.3	0.0 ± 0.0	0.679
RNFL (μm)	15.2 ± 1.8	15.4 ± 1.1	0.264
mGCL-IPL (μm)	60.8 ± 9.2	68.3 ± 7.2	0.014
IRL (μm)	215.1 ± 18.9	221.1 ± 11.6	0.095
ORL (μm)	84.3 ± 3.6	83.8 ± 1.5	0.253

Quantitative values are expressed in mean ± SD (standard deviation). BCVA—best-corrected visual acuity; RNFL—retinal nerve fiber layer; mGCL-IPL—macular ganglion cell layer–inner plexiform layer; IRL—inner retinal layer; ORL—outer retinal layer. *Student’s t-test was performed to obtain p-values.*

**Table 4:** Morpho-functional comparison between strabismus group post-surgery and control group.

	Strabismus Group Post-Surgery	Control Group	*p* Value
BCVA (logMAR)	0.0 ± 0.2	0.0 ± 0.0	0.893
RNFL (μm)	16.1 ± 2.8	15.4 ± 1.1	0.331
mGCL-IPL (μm)	66.1 ± 13.2	68.3 ± 7.2	0.127
IRL (μm)	219.2 ± 25.3	221.1 ± 11.6	0.164
ORL (μm)	84.9 ± 2.7	83.8 ± 1.5	0.431

Quantitative values are expressed in mean ± SD (standard deviation). BCVA—best-corrected visual acuity; RNFL—retinal nerve fiber layer; mGCL-IPL—macular ganglion cell layer–inner plexiform layer; IRL—inner retinal layer; ORL—outer retinal layer. *Student’s t-test was performed to obtain p-values.*

## Data Availability

All data generated or analyzed during this study are included in this article. Further enquiries can be directed to the corresponding author.
